# Effects of Computer-Aided Interlimb Force Coupling Training on Paretic Hand and Arm Motor Control following Chronic Stroke: A Randomized Controlled Trial

**DOI:** 10.1371/journal.pone.0131048

**Published:** 2015-07-20

**Authors:** Chueh-Ho Lin, Li-Wei Chou, Hong-Ji Luo, Po-Yi Tsai, Fu-Kong Lieu, Shang-Lin Chiang, Wen-Hsu Sung

**Affiliations:** 1 School of Gerontology Health Management & Master Program in Long-Term Care, Taipei Medical University, Taipei, Taiwan (R.O.C.); 2 Department of Physical Therapy and Assistive Technology, National Yang-Ming University, Taipei, Taiwan (R.O.C.); 3 Physical Medicine and Rehabilitation Department, Taipei Veterans General Hospital, Taipei, Taiwan (R.O.C.); 4 Cheng Hsin Hospital, Taipei, Taiwan (R.O.C.); 5 Department of Physical Medicine and Rehabilitation, Tri-Service General Hospital, Taipei, Taiwan (R.O.C.); 6 Department of Physical Medicine and Rehabilitation, School of Medicine, National Defense Medical Center, Taipei, Taiwan (R.O.C.); University of Glasgow, UNITED KINGDOM

## Abstract

**Objective:**

We investigated the training effects of interlimb force coupling training on paretic upper extremity outcomes in patients with chronic stroke and analyzed the relationship between motor recovery of the paretic hand, arm and functional performances on paretic upper limb.

**Design:**

A randomized controlled trial with outcome assessment at baseline and after 4 weeks of intervention.

**Setting:**

Taipei Veterans General Hospital, National Yang-Ming University.

**Participants:**

Thirty-three subjects with chronic stroke were recruited and randomly assigned to training (n = 16) and control groups (n = 17).

**Interventions:**

The computer-aided interlimb force coupling training task with visual feedback included different grip force generation methods on both hands.

**Main Outcome Measures:**

The Barthel Index (BI), the upper extremity motor control Fugl-Meyer Assessment (FMA-UE), the Motor Assessment Score (MAS), and the Wolf Motor Function Test (WMFT). All assessments were executed by a blinded evaluator, and data management and statistical analysis were also conducted by a blinded researcher.

**Results:**

The training group demonstrated greater improvement on the FMA-UE (p<.001), WMFT (p<.001), MAS (p = .004) and BI (p = .037) than the control group after 4 weeks of intervention. In addition, a moderate correlation was found between the improvement of scores for hand scales of the FMA and other portions of the FMA UE (*r* = .528, p = .018) or MAS (*r* = .596, p = .015) in the training group.

**Conclusion:**

Computer-aided interlimb force coupling training improves the motor recovery of a paretic hand, and facilitates motor control and enhances functional performance in the paretic upper extremity of people with chronic stroke.

**Trial Registration:**

ClinicalTrials.gov NCT02247674.

## Introduction

The abilities of the hands and upper extremities play important roles in daily activities. Stroke is the major leading cause of long-term disability due to paretic limbs, particularly the hands and arms [1, 2]. Clinical symptoms of stroke include reduced muscle strength, paresis, spasticity and dis-coordination (abnormal movement) of the hemiparetic upper limb [3, 4], which decrease independent performance of the activities of daily living (ADLs) and the quality of life [4–6]. Recent studies have also shown that the force control of a paretic hand is strongly associated with motor and functional performances following stroke [7, 8]. Spontaneous recovery of the movement of paretic limbs can occur; however, several studies have demonstrated that the restoration of the full functional performance of a paretic upper limb occurs in less than 15% of people following stroke, and restoration is even less likely to occur in paretic hands [9–11]. Fortunately, one study reported that the force control of paretic hands relies on visual information feedback during bimanual force tasks in people with chronic stroke [12]. Hence, it is necessary to develop effective exercise interventions that are based on recovery mechanisms that include the theories of neural plasticity and visual information feedback to improve paretic hand movement and facilitate the usage of paretic arms, which will restore functional performance and increase the independence of people with chronic stroke.

Early studies demonstrated that functional recovery and neural plasticity can be induced by active, functional or technical exercise or practice [13–16]. Therefore, in the last decade, several studies have demonstrated that active rehabilitation programs that involve bilateral movement training facilitates cortical and neurologic plasticity via three mechanism: increasing motor cortex disinhibition in the damaged hemisphere, increasing the recruitment of the ipsilateral and contralateral motor pathways in both hemispheres, and reorganizing the affected hemisphere to enhance the recovery of function in stroke patients with severe impairments [17–21]. For example, bilateral arm training with rhythmic auditory cueing (BATRAC) is one of the most commonly used training programs for improving the motor functions of the paretic upper extremities of people with stroke. The BATRAC intervention includes temporal and spatial coupling and causes interactions between both limbs [18, 21]. In this training program, each stroke subject is asked to grasp a handle with the unaffected hand while the affected hand is strapped to another handle (if the patient has severe deficits). Next, the subject simultaneously (in phase) or alternately (antiphase) pushes the handles away and pulls the handles toward the body [18, 21]. This intervention effectively improves the functional motor performances of the paretic upper limbs of sub-acute and chronic stroke patients [18, 21]. However, this effective training strategy of bilateral movement focuses only on improving paretic arm movements and is not applied to induce force control and functional recovery of the paretic hand because the affected hand is strapped to the handle individuals with severe deficits due to stroke, which is important because the functional performances of many daily activities require the participation of both hands. There are rare studies that have investigated the benefits to the paretic hand [22]. Symmetrical bilateral grip force performance is an important capability for daily activities, such as grasping a bottle or unscrewing a jar simultaneously with both hands, and involves symmetry in temporal, spatial, force and kinematic parameters (i.e., the coupling between the two hands) [23, 24]. Early studies demonstrated that bilateral movement training improves the functional recoveries of paretic upper limbs in people with stroke [18–21, 25]; however, a recent study by Kwakkel and colleagues found that modification of BATRAC did not elicit greater improvements than conventional therapy [26]. This latter finding might be because the modified BATRAC exercises focused only on wrist rotation movements and not on hand movements, did not involve time constrains for task execution, and allowed the patients to move both hands simultaneously or separately in the in-phase and anti-phase modified BATRAC exercises and extension exercises and thus did not strongly link and enforce cooperation between the two hemispheres. Furthermore, a previous study also investigated the effects of bilateral interference in a bimanual isometric force task and the relationship between the degree of interference and the asymmetries in the forces generated by each hand at different force levels in healthy individuals. The results of this study demonstrated that asymmetric force generation ratios less than 1:8 or greater than 8:1 between the two hands during a bimanual task significantly decreased the coupling effect of motor control and caused significant interference in the performances of both hands. These findings indicate that symmetrical in-phase movements are inherently stable patterns of coordination between both hands [27]. Furthermore, based on the physiology of motor control, the hand area of the primary motor cortex is larger than the representations of other body parts, which could easily be exploited to induce neural plasticity and functional recovery via an active, functional bimanual exercise program because early studies have reported that the majority of stroke survivors achieve independent ambulation after rehabilitation involving bilateral training of the lower extremities (the lower extremity area of the primary motor cortex is also larger than those of other body parts) [1]. Thus, we believed that bilateral training with visual feedback information might facilitate the recovery from impairments or the motor function (activity) of the paretic hand and might improve the motor deficits of the paretic arm and thus enhance daily functional performance via the reorganization of the affected hemisphere [17]. Therefore, the purpose of this study was to investigate the effects of bilateral movement therapy on the paretic hand and the motor control of the arm following chronic stroke. The first specific aim was to investigate the effects of training on the paretic hand after 4 weeks of bilateral movement therapy intervention in patients with chronic stroke. The second specific aim was to investigate whether the improvements in the paretic hand could facilitate the motor recovery of the paretic arm and the functional performance of patients with chronic stroke. We hypothesized that bilateral movement therapy would improve the motor recovery of the paretic hand and result in the induction of paretic arm movements and improvements in daily functional performance in patients with chronic stroke.

## Methods

### Ethics Statement

This study was approved by the Institutional Review Board of the Taipei Veterans General Hospital on Dec 7, 2012 (approval status: approved; Approval Number: 2012-11-019BY) (**S1 File**), and informed consent forms were signed by each patient. The authors confirm that all ongoing trials related to this intervention are registered. Additionally, this study was not registered (ClinicalTrials.gov) before the enrolment of participants began because such registration is not required by the national laws of Taiwan.

### Participants

**Fig 1:**
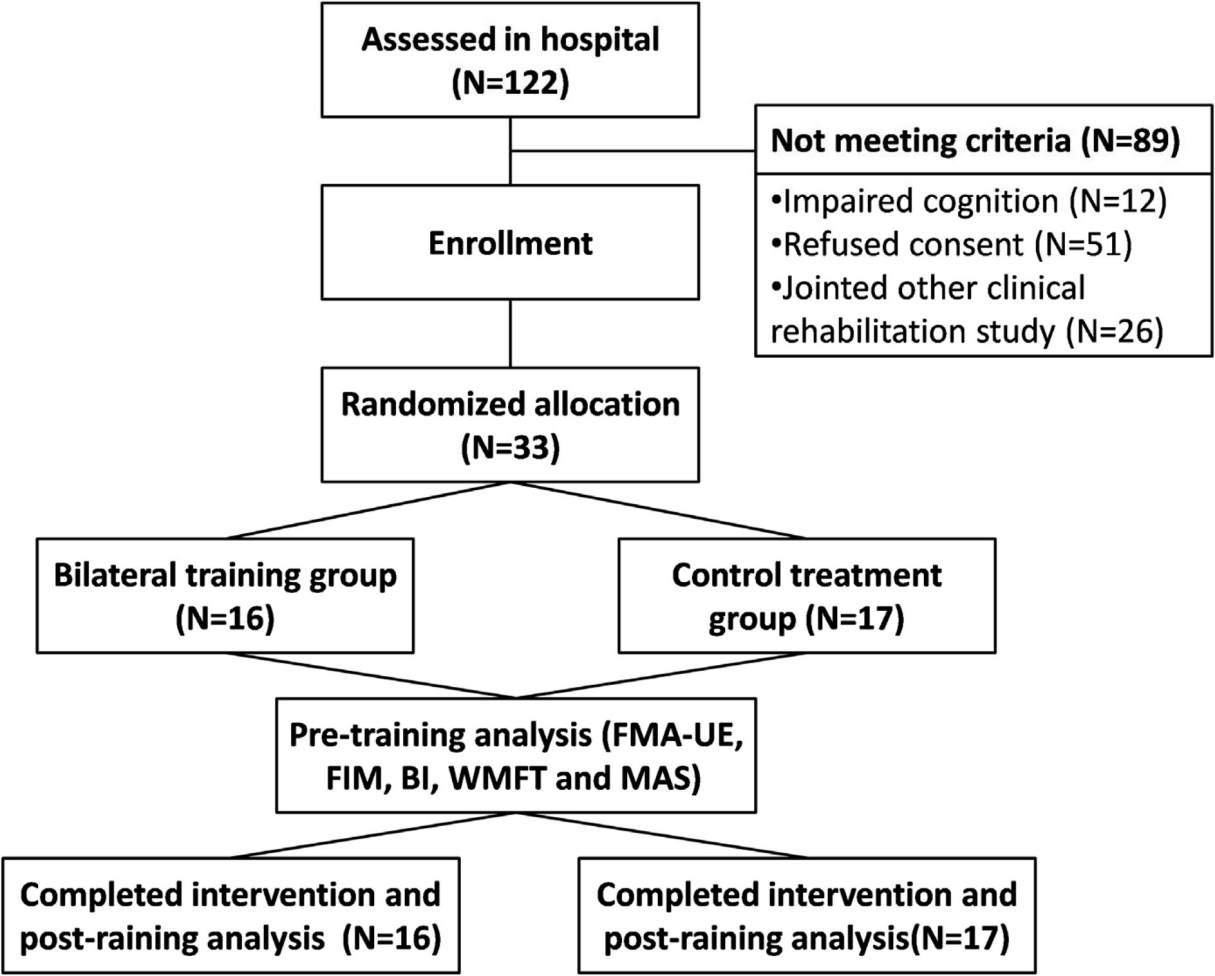
Flow diagram of the randomization procedure and the outcome measurements.

Thirty-three patients who met the criteria (mean age = 55.1±10.5, four females and 29 males, mean time since stroke onset, 23.6 months) were recruited and agreed to join the study. These patients were randomly assigned to either a bilateral isometric handgrip force training group (N = 16) or a control group (N = 17).The patients were recruited from the Taipei Veterans General Hospital between January 2013 and October 2013 (this study was approved and valid from Dec 7, 2012 to Nov 15, 2013).The flow chart of the study is presented in Fig 1. The target sample size of 32 per group was chosen to give 95% power to detect at the 5% significance level a difference of FMA score for bilateral training between the intervention and control groups(effect size = 0.842) [28]using G*Power (Version3.1.9.2).The clinical history, demographic characteristics, and baseline outcome measurements of the participants in each group are shown in Table 1. The inclusion criteria for the stroke people were as following: (1) at least 6 months since the stroke [21, 29], (2) three or fewer incidents of unilateral stroke as confirmed by collecting each participant’s medical history, (3) the ability to follow the researcher’s instructions [29], (4) the ability to flex and extend the paretic arm and hand, (5) a Modified Ashworth Score (MAS) ≦3 for the wrist and finger joints [29, 30], (6) a Mini-Mental State Examination (MMSE) score of 24 or higher[19, 21, 31], (7) no other orthopedic or neurological disorders [19], (8) Brunnstrom stage 3 or 4, and (9) no participation in other experimental rehabilitation or drug studies [19]. The exclusion criteria were as follow: (1) unstable cardiovascular conditions [21], (2) uncontrolled hypertension (190/110 mm Hg) [21], (3) severe orthopedic or pain conditions, (4) aphasia with an inability to follow the researcher’s commands, and (5) severe joint contracture of the bilateral upper extremities that would impact the movement performance of the upper extremities[21].

**Table 1:** Demographic characteristics and differences in baseline outcome measurements.

	Training group (N = 16) mean ± SD	Control group (N = 17) mean ± SD	Statistic value	p value	95% CI of the Difference
Age (y)	52.63±10.49	57.47±10.29	*t*_31_ = -1.339	.190	-12.23~2.54
BW (kg)	63.03±9.69	69.77±8.01	*t*_31_ = -2.182	.037*	-13.04~-0.44
BH (cm)	166.56±8.70	168.38±7.84	*t*_31_ = -.632	.532	-7.69~4.05
Gender (F/M)	4/12	1/16	*χ* ^*2*^_1_ = 2.343	.126	-
Lesion side (R/L hemisphere)	8/8	8/9	*χ* ^*2*^_1_ = .029	.866	-
Lesion type (hemorrhage / infarction)	7/9	11/6	*χ* ^*2*^_1_ = 1.460	.227	-
Dominant hand (R/L)	12/4	17/0	*χ* ^*2*^_1_ = 4.863	.028*	-
Months post stroke	27.75±19.04	21.82±21.66	*t*_31_ = .833	.411	-8.59~20.44
FMA-UE	35.69±15.56	38.71±19.98	*t*_31_ = -.482	.633	-15.79.75
Hand and wrist	12.38±8.06	15.12±11.04	*t*_31_ = -.881	.424	-9.64~4.16
Others	23.31±7.94	23.59±9.77	*t*_31_ = -.089	.930	-6.62~6.07
WMFT	44.25±16.84	48.47±20.42	*t*_31_ = -.646	.523	-17.56~9.11
MAS	9.19±6.21	10.53±6.70	*t*_31_ = -.596	.556	-5.93.25
BI	78.13±16.52	78.82±20.43	*t*_31_ *=* -.108	.915	-13.94~12.54

Abbreviations: y, years; BW, body weight; BH, body height; F, female; M, male; R, right; L, left; FMA-UE, upper-extremity portion of the Fugl-Meyer assessment; WMFT, Wolf Motor Function Test; MAS, Motor assessment scale; BI, Barthel Index.

* Significant difference P < .05.

### Study design

This study used a randomized control group design. All participants were randomly assigned to either a bilateral isometric handgrip force training group or a control group. The investigator used a computer generated random number sequence for randomization. After recruitment and coding by a clinical physician, the randomization procedure was performed by a researcher using a randomization computer program to allocate a sequence condition code of 1 or 2 to each subject (1 indicates the training group, and 2 indicates the control group). For each patient, all other routine rehabilitation programs that did not involve bilateral UE training, including physical therapy, occupational therapy and speech therapy, proceeded as usual. To provide consistent interventions, a single certified physical therapist who was trained in the administration of bilateral isometric handgrip force training administered the protocols that were written by the primary investigators at the Taipei Veterans General Hospital. Before and after the 4-week intervention period, clinical measures were evaluated by another certified, trained physical therapist who was blinded to the participant group at the Taipei Veterans General Hospital. The analyses of the outcome measurements were performed at National Yang-Ming University by a statistician who was also blinded to the participant groups.

### Intervention protocols

Therefore, we developed a novel bimanual training program (i.e., bilateral isometric handgrip force training) with visual feedback and symmetric force generation, based on the concept of bilateral movement therapy. The bilateral isometric handgrip force training consisted of 30 minutes of training 3 days per week for 4 weeks, for a total of 12 sessions. For the bilateral isometric handgrip force training group, each subject sat in a comfortable chair (the height was adjusted for safety) facing the operation table, which contained a training apparatus and an LCD screen. The bilateral upper limbs were positioned such that the grip force training exercise could be executed comfortably. The forearms were fixed onto forearm supports to avoid abnormal compensation movements that could affect the rehabilitation training. The training position placed the shoulder joints at 30°–40° adduction in the horizontal plane and 40°–50° flexion in the sagittal plane, with the elbows at 100°–110° flexion and the wrists in neutral position such that the fingers could easily be flexed to 90° to hold the dynamometers. When necessary, Velcro was used to prevent abnormal compensatory movements. The apparatus was custom made by the Ya-May Company (Taipei, Taiwan) and consisted of two dynamometers, two bow-shaped handles made from polyvinyl cylinders, four rotary potentiometers, and two forearm supports.

Bilateral isometric handgrip force training imitates the movements of the functional activities of daily living, which require strength, coordination and control of the hands [32, 33]. This training mimics the daily activities that require grip strength coordination and thus might improve the recovery of the paretic hands [23, 34].The grip forces were applied simultaneously and continuously by the two hands. After verbal cueing by the trainer, the individual gradually increased or decreased their grip strengths with both hands to track the trajectory of the targeted force. The trajectory was generated by a computer program (LabVIEW 2009). During this process, the grip strength and trajectory of each hand were displayed in real-time on an LCD monitor to provide direct visual feedback. In addition, before the bilateral isometric handgrip force training, the MVC test was used to determine the strengths of the maximal contractions of both of the hands of the stroke patients. Each subject was asked to grasp the dynamometer with their maximum grip force and the MVC testing protocol followed previously reported methods [29]. A value of 40% MVC for the hemiparetic hand grip strength was set as the target force during the training. Furthermore, each subject was trained via the repetitive generation of bilateral hand grip forces to match the targeted forces and simultaneous (in-phase) the relaxation. The required hand grip forces were indicated by a computer program that was set at the preferred speed (the increases in speed ranged from 0.001%MVC/s to 0.03%MVC/s). To prevent muscle fatigue, the training blocks were repeated three times (each block was 15 minutes in duration) with a 5 minute resting intervals between blocks for a total of 30 minutes of bilateral training for each participant. This training protocol was modified from those of previously studies [18, 19, 21, 22, 29]. Furthermore, the patients were also encouraged to smoothly generate the grip force with their paretic hand during the training period by increasing the strength-to-the-target force. In the control groups, routine clinical rehabilitation was maintained for the duration of the study period. This rehabilitation included strengthening, stretching, practicing of functional tasks, and coordination and weight bearing training of the hemiparetic upper limb. We also provided education and training consultations regarding factors such as muscle strength exercise and task-oriented exercise for the unilateral paretic upper extremity for the subjects in the control group.

### Outcome measurements

The pre-specified primary outcome measure was clinical motor assessment and secondary outcome measures were functional performance assessments. All outcome measurements were collected upon entry into the study (baseline) and 4 weeks after the completion of the training program. The motor assessment was the upper-extremity portion of the Fugl-Meyer assessment (FMA-UE). FMA is the most widely used assessment of the motor impairments of the paretic limbs of persons with stroke [35–38]. The functional performance evaluations were the Barthel Index (BI), the Wolf Motor Function Test (WMFT) and the Motor assessment scale (MAS). The BI is the most widely used method for measuring the independence of daily activities in clinical settings. The WMFT is a time-based method with high inter-rater reliability and test-retest reliability that was designed to evaluate the functional disabilities of the upper limbs of people with stroke [35, 39–41]. The MAS was developed to evaluate the progress in motor performance following stroke and is highly reliable[36, 42, 43]. The demographic data and medical histories were also collected prior to the intervention.

### Statistical Analyses

The FMA UE scales, BI, WMFT and MAS data regarding the functional abilities of the impaired upper limbs before and after the intervention were analyzed with two-way mixed analyses of variance (ANOVA). The factors in the 2×2 ANOVA were the between-subject variable of group (the bilateral isometric handgrip force training group and the control group) and the within-subject variable of time (pre-intervention vs. post-intervention). Furthermore, the relationships between the improvements in the FMA, FMA UE, BI, WMFT and MAS scores were calculated with Spearman's correlation coefficients. Values of *r* between 0.7 and 0.89 indicate a strong correlation; *r* values between 0.5 and 0.69 indicate moderate correlations; and *r* values between 0.3 and 0.49 indicate weak correlation. The alpha level for statistical significance was set at 0.05. SPSS version 16.0 statistical software was used.

## Results

### Differences in Baseline Performance

There were no statistically significant differences between the training and the control groups in the baseline clinical data or outcome measurements of the FMA-UE (35.7±15.6 vs. 38.7±20.0; p = .142), BI (78.1±16.5 vs. 78.8±20.4; p = .223), Wolf (44.3±16.8 vs. 48.4±20.4; p = .423) and MAS (9.2±6.2 vs. 10.5±6.7; p = .573) (Table 1). Therefore, the conditions of movement and functional performances were similar for both groups. Furthermore, a review of the routine clinical therapy records (i.e., physical and occupational therapy) during the study period revealed that no patients had previously undergone bilateral training of the upper extremities.

### Changes in Upper-Limb Movement and Related Differences in Functional Performance

The training group exhibited significantly improved motor control and functional performance of the paretic upper extremity after the 4-week intervention as measured by the FMA-UE (p < .001), BI (p = .001), Wolf (p < .001) and MAS (p = .001) scores (Table 2). The control group exhibited no significant improvements in paretic upper extremity movement in terms of the FMA-UE (p = .067), BI (p = .231) and Wolf (p = .550) scores. The control group did exhibit an improvement in the MAS score (p = .044) (Table 2). Furthermore, compared with the control group, the bilateral grip force training task significantly improved the recovery of paretic arm and hand movements as indicated by the FMA-UE (F_(1, 30)_ = 31.359,p<0.01), BI (F_(1, 30)_ = 5.557,p = 0.037), Wolf (F_(1, 30)_ = 18.557,p<0.01) and MAS (F_(1, 30)_ = 281.264,p<0.01) scores (Table 2). Furthermore, compared with the control group, we observed a significant moderate correlation (*r* = .528, p = .018) between the scores for the hand scales of the FMA and the other portions of the FMA UE. There was also a strong correlation between the improvement in the FMA score and the score for the other portions of the FMA UE (*r* = .825, p < .001) in the training group (Table 3). Additionally, there were a significant moderate correlations (*r* = .596, p = .015; *r* = .625, p = .010) between the improvements in the scores for the hand scales on the FMA, the FMA UE and the MAS in the training group (Table 3).

**Table 2:** Changes in motor and functional measurements in the training group and the control group.

	Training group (N = 16) Mean ± SD						Control group (N = 17) Mean ± SD						Improvement difference between two groups Mean difference± SE		
	Pre- treatment	Post- treatment	Differences (95%CI)	p value	F-value	η2	Pre- treatment	Post- treatment	Differences (95%CI)	p value	F-value	η2	Differences (95%CI)	t value	p value
**FMA-UE**	35.69±15.56	46.75±16.04	11.06±5.63(8.064~14.061)	.000*(1.063x10^-6)	61.842	0.805	38.71±19.98	40.41±19.90	1.71±3.58 (-.137~3.549)	.067	3.851	.194	9.36±1.63 (6.028~12.685)	*t*_31_ = 5.734	.000** (2.624x10^-6)
Hand and wrist	12.38±8.06	19.31±8.52	6.94±3.55(5.046~8.829)	.000*(1.140x10^-6)	61.136	.803	15.12±11.04	16.00±11.74	.88±2.12 (-.207~1.971)	.105	2.951	.156	6.06±1.01 (3.995~8.115)	*t*_31_ = 5.995	.000** (1.246x10^-6)
Other portion	23.31±7.94	27.44±8.05	4.13±3.34(2.343~5.90)	.000*(1.800x10^-4)	24.344	.619	23.59±9.77	24.41±9.45	.82±3.25 (-.845~2.492)	.311	1.095	.064	3.30±1.15 (.962~5.641)	*t*_31_ = 2.878	.007**
**WMFT**	44.25±16.84	51.19±17.48	6.94±4.34(4.623~9.252)	.000*(1.217 x10^-5)	40.825	.731	48.47±20.42	49.06±20.94	0.59±3.97 (-1.453~2.629)	.550	.373	.023	6.35±1.45 (3.398~9.300)	*t*_31_ = 4.388	.000** (1.231x10^-4)
**MAS**	9.19±6.21	12.00±5.50	2.81±2.56(1.447~4.178)	).001* (5.257 x10^-4	19.286	.562	10.53±6.70	11.18±6.51	0.65±1.22 (.019~1.275)	.044*	4.768	.230	2.165±.692 (.754–3.577)	*t*_31_ = 3.130	.004**
**BI**	78.13±16.52	91.25±13.48	13.13±13.52(5.918~20.332)	.001*(1.475 x10^-3)	15.068	.501	78.82±20.43	82.35±19.93	3.53±11.69 (-2.483~9.542)	.231	1.548	.088	9.596±4.393 (.635–18.556)	*t*_31_ = 2.184	.037

Abbreviations: FMA-UE, upper-extremity portion of the Fugl-Meyer assessment; WMFT, Wolf Motor Function Test; MAS, Motor assessment scale; BI, Barthel Index.

* Significant difference P < .05.

** Significant difference P < .01.

**Table 3:** Correlations between the recovery of the paretic hand and changes in motor performance outcome measurements.

	Training group (N = 16)				Control group (N = 17)			
	Hand score of FMA-UE	Sig. (2-tailed)	FMA-UE	Sig. (2-tailed)	Hand score of FMA-UE	Sig. (2-tailed)	FMA-UE	Sig. (2-tailed)
**Other portion of FMA UE**	.528	.018*	.825	.000*	.032	.451	.653	.004*
**FMA UE**	.835	.000*	-	-	.578	.015*	-	-
**BI**	.432	.095	.238	.374	.414	.099	.280	.277
**WMFT**	.330	.213	.398	.126	-.051	.846	.118	.651
**MAS**	.596	.015*	.625	.010*	.404	.108	.317	.215

Abbreviations: FMA-UE, upper-extremity portion of the Fugl-Meyer assessment; BI, Barthel Index; WMFT, Wolf Motor Function Test; MAS, Motor assessment scale.

* Significant difference P < .05.

## Discussion

Most daily activities require complex bilateral hand coordination[6]. Therefore, recovery of the motor function of paretic hands is critical for stroke patients. This is the first study to demonstrate the treatment effects of bilateral training approaches on the paretic hands of participants with chronic stroke. We examined the effects of bilateral isometric handgrip force training on motor control and functional performance outcomes, including the FMA UE, BI, WMFT and MAS scales in people with chronic stroke. Furthermore, we also investigated whether the improvements in the FMA UE, BI, WMFT and MAS scores were facilitated by and related to the recovery the function of the paretic hand.

### Improvements in the Motor Control and Functional Performance of the paretic upper limb

Training significantly improved the motor control and functional abilities of the paretic hands, arms and upper extremities as measured with the FMA UE, WMFT, MAS and BI. For example, the motor improvements of the paretic hands of the training group were greater than those in the control group as indicated by the 9.4-point greater reduction the score for the hand scales of the FMA. This findings is consistent with those of previous studies that have demonstrated that bilateral movement training (i.e., the executing simultaneous execution of activities or specific functional tasks with both upper extremities) reorganizes the inhibition and dis-inhibition of neural excitation in both hemispheres, which facilitates neural activation in the damaged hemisphere and promotes motor recovery in the paretic upper extremity [17–21]. However, the effects of training on the motor control and functional performance of the paretic hand have rarely been discussed in previous studies.

Previously studies have demonstrated that bilateral movement training might facilitate neural plasticity in stroke patients in the following manners: (1) facilitating cortical neural plasticity and the formation of new connections (reorganization) between neurons in the damaged motor cortex, (2) normalizing abnormal disinhibition in the motor cortex and excess inhibition delivered through the corpus callosum between the damaged and un-damaged hemispheres, (3) enhancing neuronal excitation in the corticospinal pathways ipsilateral to the unaffected hemisphere, and (4) improving supplementary motor area (SMA) function by supplementing the motor control of the paretic upper limbs [17, 25, 44–47]. These recovery mechanisms explain why the treatment improved the motor control and functional performance of the paretic upper extremities following chronic stroke. The first mechanism was described by Rouiller and colleges in 1998. Their study showed that infant monkeys with damage to the motor cortex were able to re-organize motor pathways to healthy peri-lesional neurons of the injured hemisphere 2–6 years after the lesion[46]. Regarding the second mechanism, early studies using transcranial magnetic stimulation (TMS) revealed an imbalance in the interactions between the hemispheres that includes a decreased in the ability of the motor cortex to disinhibit the hemiparetic hemisphere and an increase in intracortical inhibition in the healthy hemisphere in people following stroke [48]. Abnormal intracortical inhibition is decreased when people execute synchronized movements of the bilateral upper extremities [47]. The third mechanism is the most accepted explanation of the bilateral treatment effect; however, an intact cortical-spinal tract is necessary for motor improvements. The last mechanism remains controversial because changes in neural excitation in the motor cortex as detected by fMRI vary depending on the training task, intensity, study protocol and evaluation condition [49–51].

### Correlation of motor recovery in the paretic hand and arm and functional performance

The patients in the training group reported increased use of the paretic upper extremity due to a reduction in the impairment of the paretic hand during the study period. Indeed, we demonstrated that improvements in the paretic hand induced motor recovery and improved functional performance in the paretic arm, and we demonstrated a significant recovery of dexterity and functional performance in the paretic upper limb as measured with the WMFT, MAS and BI. Therefore, this phenomenon might be helpful for understanding the relationship between the motor improvements in the paretic arms and hands. Indeed, we analyzed the outcome measurement data from the training group and found that the recoveries indexed by the scores for hand scales of the FMA and the scores for the other portions of the FMA UE (i.e., the arm portion) exhibited a significant moderate correlation. We also observed a strong correlation between the improvements in the FMA scores and improvements in portions of the FMA UE. Additionally, we observe significant moderate correlations between the improvements in the scores for the hand scales of the FMA, FMA UE and MAS in the training group. These findings indicate that bilateral isometric handgrip force training not only improves the motor recovery of the paretic hands but also facilitates the movements of the paretic arms and forearms. These findings have never been reported in previous study. Previous studies have demonstrated that bilateral movement therapy can promote motor recovery in the paretic upper extremities of chronic stroke patients [3, 18–21, 25]. However, comparisons between other bilateral movement training studies and this study are difficult due to the different interventions used and the different intensities of training of the participants with chronic stroke that demonstrated improvements in motor and functional outcomes following bilateral task interventions. We suppose there are five active reasons for these differences in the motor recoveries between the groups. First, compare with previous studies, the bimanual exercise in this study focus on bilateral isometric handgrip force generation with both hands with visual feedback based on the concept of bilateral movement therapy and did not focus only on wrist rotation [26]. Second, the training protocol of the present study focused not only on repetitive bilateral hand grip strength generation to match target forces but also on the simultaneous relaxation of the paretic hand during the task. Third, all of the subjects were asked to actively position their bilateral shoulder joints at 30°–40° adduction in the horizontal plane and 40°–50° flexion in the sagittal plane with the elbow at 100°–110° flexion during the bilateral training; this position in helpful for preventing abnormal flexion synergy patterns in the paretic upper limb and might also have improved the performances of the paretic arms and forearms. Fourth, each subject in the training group traced target forces with repetitively generated hand grip tension and relaxation within time limitations (the increase in speed ranged from 0.001%MVC/s to 0.03%MVC/s) that were controlled by a computer program. Fifth, compared with the modified BATRAC exercise, the training protocol of the study focused on linking and promoting cooperation between the hemispheres. We also suggest that this bilateral movement training approach to improving the motor recoveries and functional performances of the paretic hands and arms might be better than conventional rehabilitation approaches for people with chronic stroke. However, there are a few limitations to this study that should be considered. This trial was not double-blinded and lacked follow-up data to indicate whether the effects of training persisted. This clinical study had a small sample size and consequently low power because of strict inclusion or exclusion criteria and difficult task execution requirement thus might not exactly reflect effects of the treatment in the general population of patients with stroke. However, the strength of evidence against the null hypothesis of no effect is strong because the p value achieved statistically significant and the 95% CI is far from zero as well; therefore the lower power may not seriously undermine the credibility of the result. The study design and the fact that the intervention did not include a dose effect are also limitations. Therefore, future work should address and clarify the optimal dose and intervention protocols for bilateral movement training. Additionally, this study was not registered before the enrollment of participants began because such registration is not necessary according national law in Taiwan.

## Conclusion

Bilateral isometric handgrip force training improves the motor recoveries and the functional abilities of the hemiparetic hands and arms of patients with chronic stroke. We demonstrated that facilitations of the use and movement of the hemiparetic arm and increases in the functional performance of the upper extremity were associated with improvements in the motor recoveries of the hemiparetic hands of stroke survivors.

## Supporting Information

S1 CONSORT Checklist CONSORT Checklist. (DOC)

S1 File Ethics committee approvement. (PDF)

S2 File Clinical Trial Registration. (PDF)

S3 File Changes in FMA score in both groups before and after intervention. (JPG)

S4 File Changes in WMFT score in both groups before and after intervention. (JPG)

S5 File Changes in MAS score in both groups before and after intervention. (JPG)

S6 File Changes in BI score in both groups before and after intervention. (JPG)

S1 Protocol Study protocol (English version). (DOCX)

S2 Protocol Study protocol (Chinese version). (DOCX)
