# Supplementary material for: Effects of Computer-Aided Interlimb Force Coupling Training on Paretic Hand and Arm Motor Control following Chronic Stroke: A Randomized Controlled Trial
Source: PLoS One. 2015 Jul 20;10(7):e0131048. doi: 10.1371/journal.pone.0131048 (PMC4507879; doi:10.1371/journal.pone.0131048)
Supplement: S1 File — (PDF) [file pone.0131048.s002.pdf]

行政院國軍退除役官兵輔導委員會台北榮民總醫院

TAIPEI VETERANS GENERAL HOSPITAL, VAC

201 SHIH-PAI ROAD, SEC. 2
TAIPEI, TAIWAN 11217
REPUBLIC OF CHINA
TEL: (886)-2-2871-2121(30 LINES)

同 意 臨 床 試 驗 證 明 書

查國立陽明大學物理治療暨輔助科技系宋文旭助理教授主持之「雙手握力協調訓練對於中風患者偏癱上肢復健成效」（本院 IRB 編號：2012-11-019BY）臨床試驗案，業經本院 101 年 11 月 16 日人體試驗委員會(二)第 34 次會議審查通過，有效期限至 102 年 11 月 15 日，特此證明。

計畫主持人須於到期前 6 週內提出展延計畫之申請，本案須經本院人體試驗委員會通過後，方可繼續執行。（凡需送衛生署審核之計畫案件，須取得衛生署審核同意函後方可開始執行）

人體試驗委員會 Institutional Review Board V. G. H., TAIPEI, VAC

台北榮民總醫院
人體試驗委員會
主任委員
何善台

中 華 民 國 1 0 1 年 1 2 月 7 日

Dec 7, 2012

To Whom It May Concern:

RE: Effects of bilateral handgrip coordination training on upper limb motor recovery after stroke

Principal Investigator: Wen-Hsu Sung, Ph.D.
Co-Investigator: Po-Yi Tsai, M.D.
Protocol No:
Version date of documents:

1. Protocol Version: Version 1, Date: 2012/10/12
2. Synopsis: Version 1, Date: 2012/10/12
3. Informed Consent Form: Version 2, Date: 2012/11/11
4. DSMP: Version 2, Date: 2012/11/11
5. Advertisement：Version 2, Date: 2012/11/11

VGHIRB No.: 2012-11-019BY

According to the written operating procedures, GCP, and the applicable regulatory requirements, this study project is approved by the Institutional Review Board of Taipei Veterans General Hospital. The board is organized under, and operates according to International Conference on Harmonisation (ICH) / WHO Good Clinical Practice (GCP) and the applicable laws and regulations.
This approval is valid for 1 year till Nov 15, 2013. The principal investigator is required to submit the application for extension 6 weeks before the expiration date. (If indicated by the regulations and laws, this project should be taken after the approval of Department of Health, R.O.C.)

S-T. Ho

Shung-Tai Ho, M.D.
Chairman
Institutional Review Board
Taipei Veterans General Hospital
Taiwan, R.O.C.
