# Supplementary material for: Effects of Computer-Aided Interlimb Force Coupling Training on Paretic Hand and Arm Motor Control following Chronic Stroke: A Randomized Controlled Trial
Source: PLoS One. 2015 Jul 20;10(7):e0131048. doi: 10.1371/journal.pone.0131048 (PMC4507879; doi:10.1371/journal.pone.0131048)
Supplement: S2 File — (PDF) [file pone.0131048.s003.pdf]

ClinicalTrials.gov PRS
Protocol Registration and Results System

## Protocol Registration Preview

Close

# Bilateral Movement Training for People With Stroke

**This study has been completed.**

| | |
|---|---|
| Sponsor: | Chueh-Ho Lin |
| Collaborators: | National Yang Ming University |
| Information provided by (Responsible Party): | Chueh-Ho Lin, National Yang Ming University |
| ClinicalTrials.gov Identifier: | NCT02247674 |

## Purpose

The investigators hypothesized that bilateral handgrip force training would result in significant improvements in paretic hand, arm movements and daily functional performances. In order to investigate whether the improvement of paretic hand could facilitate the motor recovery of paretic arm and functional performances, the investigators also hypothesized that motor recovery and functional performances improvements of paretic arm and hand have strongly correlation.

| Condition | Intervention | Phase |
|---|---|---|
| Stroke<br>Cerebrovascular Accident<br>Cardiovascular Disease<br>Brain Injury | Procedure/Surgery: Bilateral movement training | N/A |

Study Type: Interventional
Study Design: Treatment, Parallel Assignment, Double Blind (Subject, Outcomes Assessor), Randomized, N/A

Official Title: Effects of Computer-aided Interlimb Force Coupling Training on Paretic Hand and Arm Motor Control Following Chronic Stroke

**Further study details as provided by Chueh-Ho Lin, National Yang Ming University:**

Primary Outcome Measure:

- Upper Extremity motor control of Fugl-Meyer Assessment (FMA-UE) [Time Frame: before and after 4 weeks intervention] [Designated as safety issue: No]

Outcome measure was assessed before and after 4 weeks intervention.

Secondary Outcome Measures:

- Barthel Index (BI)   [Time Frame: before and after 4 weeks intervention] [Designated as safety issue: No]
  Outcome measure was assessed before and after 4 weeks intervention.
- Motor Assessment Score (MAS)   [Time Frame: before and after 4 weeks intervention] [Designated as safety issue: No]
  Outcome measure was assessed before and after 4 weeks intervention.
- Wolf Motor Function Test (WMFT)   [Time Frame: before and after 4 weeks intervention] [Designated as safety issue: No]
  Outcome measure was assessed before and after 4 weeks intervention.

Enrollment: 33
Study Start Date: November 2012
Study Completion Date: October 2013
Primary Completion Date: May 2013

| Arms | Assigned Interventions |
| --- | --- |
| Placebo Comparator: Education & training consultation<br>Education and training consultations were provided for subjects in control group during study period. | |
| Experimental: Bilateral movement training<br>Exercise training of bilateral isometric handgrip force training group consisted of 30 minutes of bilateral isometric handgrip force training 3 days per week for 4 weeks (total 12 sessions) | Procedure/Surgery: Bilateral movement training<br>Exercise training of bilateral isometric handgrip force training group consisted of 30 minutes of bilateral isometric handgrip force training 3 days per week for 4 weeks (total 12 sessions)<br>Other Names:<br>• Bilateral movement training<br>• Coupling movement |

Objective: To investigate the training effects of interlimb force coupling training on

paretic hand and upper extremity outcomes in patients with chronic stroke; to analyze the relationship between motor recovery of paretic hand, arm and functional abilities.

Design: A double-blind randomized controlled trial with outcome assessment at baseline and after 4 weeks intervention.

Setting: Department of physical medicine and rehabilitation in Taipei Veterans General Hospital.

Participants: Thirty-three subjects (mean age = 55.1 ± 10.5 y/o) with chronic stroke were recruited and randomized assigned to training (n=16) and control group (n=17).

Interventions: Interlimb force coupling training task included different gripforce generation on the both hands.

Main Outcome Measures: Barthel Index (BI), and the upper extremity motor control of Fugl-Meyer Assessment (FMA-UE), Motor Assessment Score (MAS), and the Wolf Motor Function Test (WMFT). All assessment was executed by a blinded evaluator, and data management and statistical analysis was also conducted by a blinded statistic researcher.

## Eligibility

Ages Eligible for Study: 28 Years to 81 Years
Genders Eligible for Study: Both
Inclusion Criteria:

1. at least 6 months since stroke
2. three or fewer incidents of unilateral stroke confirmed by taking the participant's medical history ability to follow researcher's instructions
3. ability to flex and extend the paretic arm and hand
4. Modified Ashworth Score (MAS) ≦3 for wrist and finger joints
5. Mini-Mental State Examination (MMSE) score should 24 or higher
6. no other orthopedic neurological disorders
7. Brunnstrom stage 3 or 4
8. no joint in other experimental rehabilitation or drug studies 21

Exclusion Criteria:

1. unstable cardiovascular conditions
2. uncontrolled hypertension (190/110 mm Hg)
3. severe orthopedic or pain conditions
4. dementia (Mini-Mental State Examination score < 22)
5. aphasia with inability to follow researcher's commands
6. severe joint contracture of bilateral upper extremities that would impact the movement performances of upper extremities

## Contacts and Locations

### Locations

#### Taiwan

Taipei Veterans General Hospital

Taipei, Taiwan, 112

**Investigators**

| | | |
|---|---|---|
| Study Chair: | Wen-Hsu Sung, PhD | Department of Physical Therapy and Assistive Technology, National Yang-Ming University |

## More Information

Responsible Party: Chueh-Ho Lin, Dr., National Yang Ming University
Study ID Numbers: NYMU20140829
Health Authority: Taiwan: Institutional Review Board

Close

U.S. National Library of Medicine | U.S. National Institutes of Health | U.S. Department of Health & Human Services
