# Supplementary figures and images for: Effects of Computer-Aided Interlimb Force Coupling Training on Paretic Hand and Arm Motor Control following Chronic Stroke: A Randomized Controlled Trial
Source: PLoS One. 2015 Jul 20;10(7):e0131048. doi: 10.1371/journal.pone.0131048 (PMC4507879; doi:10.1371/journal.pone.0131048)

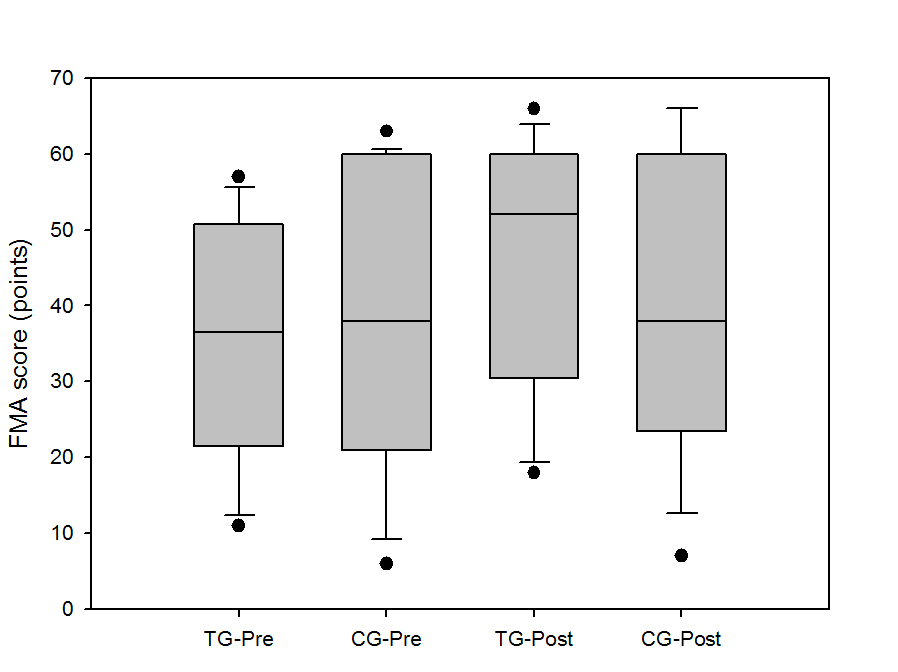

Supplement: S3 File — (JPG) [file pone.0131048.s004.JPG]

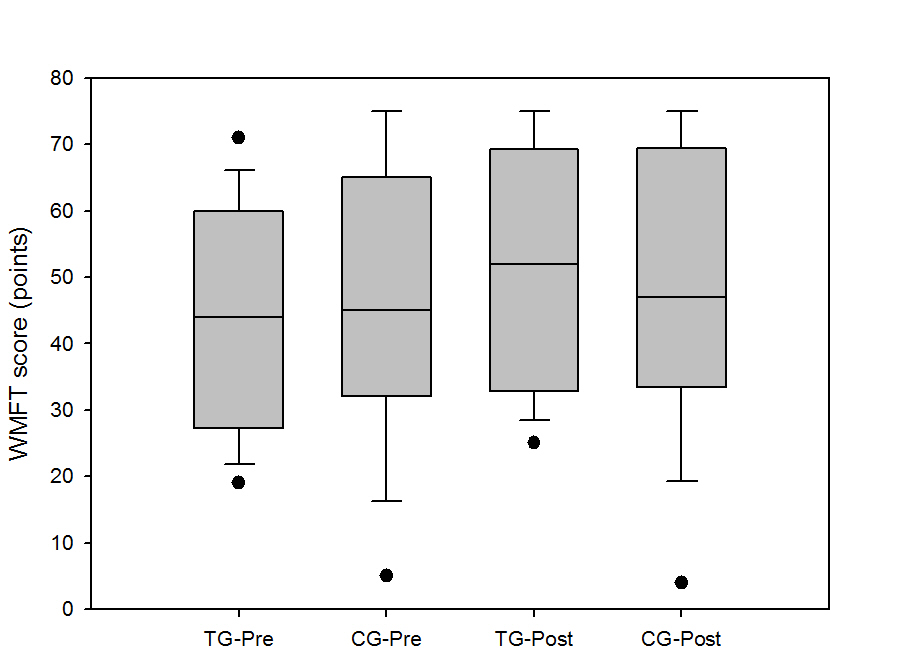

Supplement: S4 File — (JPG) [file pone.0131048.s005.JPG]

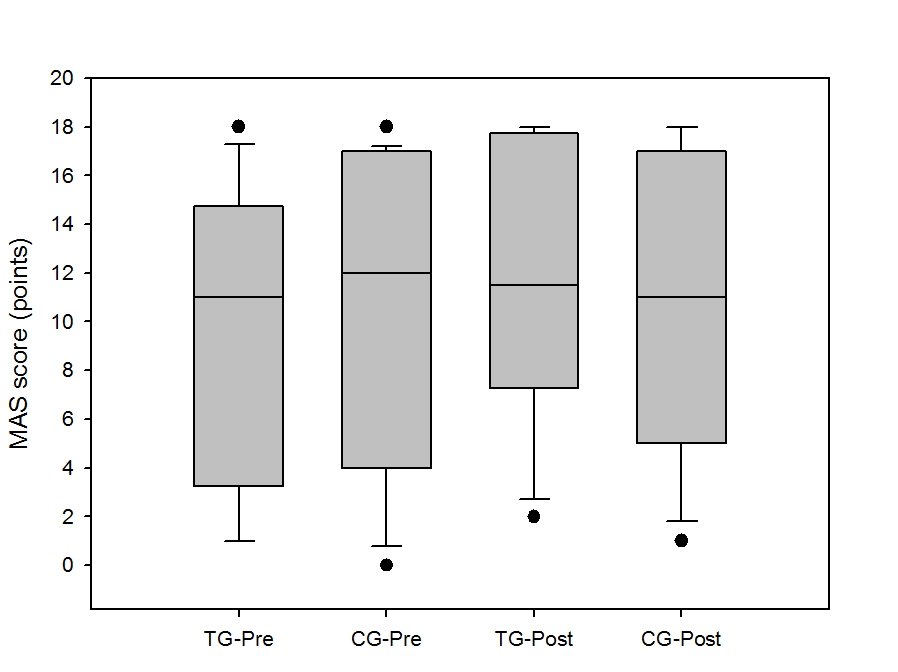

Supplement: S5 File — (JPG) [file pone.0131048.s006.JPG]

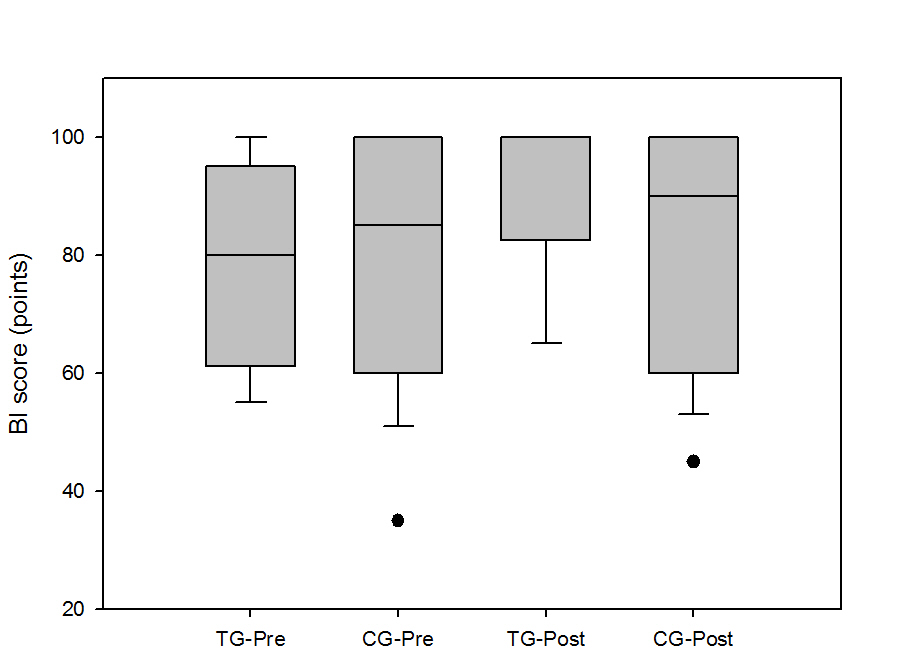

Supplement: S6 File — (JPG) [file pone.0131048.s007.JPG]
