# Supplementary material for: Effects of Computer-Aided Interlimb Force Coupling Training on Paretic Hand and Arm Motor Control following Chronic Stroke: A Randomized Controlled Trial
Source: PLoS One. 2015 Jul 20;10(7):e0131048. doi: 10.1371/journal.pone.0131048 (PMC4507879; doi:10.1371/journal.pone.0131048)
Supplement: S1 Protocol — (DOCX) [file pone.0131048.s008.docx]

**Investigation of two-handed coordination training for stroke patients suffering from upper extremity hemiplegia**

**Study protocol**

This study will analyze and evaluate hand grip strength and bilateral coordination of the upper limbs of stroke victims, and uses a computer-assisted training model to explore the possibility of effective recovery of hemiplegic limb function while promoting coordination and control of both hands. Analysis results confirm that bilateral upper limb training is effective in restoring hand function. In addition, this study employs commonly-used clinical assessment scales for upper limb function to measure progress in the recovery of hemiplegic upper limb function after training. Comparative analysis of the system parameters is used for related factor analysis to confirm the parameters’ discriminatory value and predictive value for the recovery of hemiplegic limb function. A double-blind randomized control trial will be used to assess baseline values prior to training and to evaluate the effectiveness of training four weeks following intervention.

1. **Subject screening conditions**

This study will recruit sixty people who have suffered a stroke at least six months previous and are either undergoing physical therapy or are being treated by neurologists. Patients will be recruited according to the following criteria: (1) physician diagnosis of vascular lesions caused by cerebral embolism or bleeding; (2) the patient had suffered fewer than three stroke incidents, and without other serious complications which significantly affected upper limb function; (3) stroke had occurred at least six months previously, and the patient is in stable condition (Naik et al., 2011); (4) stroke rehabilitation had already achieved Brunnstrom Stage 3 or above; (5) patients were able to understand experimental processes and related precautions (MMSE≧23) (Folstein et al., 1975); (6) Modified Ashworth Score (MAS) ≦3 for the horizontal shoulder abduction, adduction, elbow flexion, extension, finger flexion and extension (Bohannon et al., 1987); (7) patients are able to bend and straighten their fingers without assistance (Naik et al., 2011); (8) patients can sit unassisted for 30 minutes of rehabilitation exercise; and (9) patients can understand conditions for participation and give consent. Criteria for exclusion from the study included: (1) excessive upper limb muscle tone and inability to conduct isolative movements; (2) patients suffered from unilateral visual hemiansopsia or significant unilateral hemineglect which significantly impacted bilateral arm movement; (3) uncontrollable hypertension (190/110 mm Hg) or unstable cardiovascular conditions; (4) significant accompanying vestibular, cerebellar or other movement disorders; (5) severe orthopedic or other trauma which could result in pain or other symptoms during rehabilitation activity; (6) stroke-induced cognitive impairment or aphasia which could limit effective communication; (7) other neurological or mental illness, or joint contracture which would impact upper limb movement during the experiment.

1. **Primary outcome measurements**

In addition to measuring hemiplegic limb and grip performance, this study also assesses kinematic parameters for bilateral hand symmetry and upper limb movement stability using relevant clinical measurements and assessment tools for control assessment. Bilateral grip strength control evaluation includes: 1) Bilateral Handgrip Force Coordination Timing; (BHF-CT); 2) Bilateral Handgrip Force Stable Value (BHF-SV); 3) Dynamic Force Stable Value (DFSV); and Dynamic Force Stable Index (DFSI). In addition to using these parameters to evaluate bilateral coordination, this study uses relevant clinical assessment scales as control tools, such as the Motor Assessment Scale; (MAS) (Sabari et al., 2005; Blennerhassett et al., 2008), the Fugl-Meyer Assessment (Sabari et al., 2005; Lin et al., 2009), the Wolf Motor Function Test (WMFT) (Blennerhassett et al., 2008; Wolf et al., 2001; Morris et al., 2001) to assess bilateral hand motor function in stroke victims. Finally, the parameters used in the proposed system will be cross-analyzed against clinical assessment scales to confirm the effectiveness of the parameters in assessing the recovery of hand function in stroke victims.

1. **Bilateral grip coordination and control assessment**

All subjects will be asked to complete two different tasks for the contralateral and hemiplegic hands: the maximal voluntary contraction (MVC) test and the bilateral hand grip control task. The combined visual feedback provided by these bilateral grip force control tasks will include maximal voluntary hemiplegic hand target grip strength targets of 10%, 20% and 40%, using the hemiplegic hand’s maximum grip strength as the target for bilateral task implementation, thus preventing negative interference from impacting the assessment results and reducing the performance of the healthy hand (Lewis et al., 2001; Steenbergen et al., 1996).

The MVC test will mainly be used before the test task to understand the difference in the maximum voluntary grip strength of the patient’s paretic and nonparetic hands. Subjects will first be placed in a comfortable position and then given verbal cues (“begin” and “stop”) to exert their maximum grip strength on the system’s metal handle for a minimum of 6 second s (Coombes et al., 2008; Vaillancourt et al., 2003). MVC values are defined as the greatest value of three separate tests (measured for seconds 2~6 for each test) (Bigland-Ritchie et al., 1983) separated by a rest interval of 60 seconds to prevent muscle fatigue from impacting performance results (Kent-Braun et al., 1999; Shinohara et al., 2003). Once the MVC values are obtained, the bilateral grip control task will be implemented.

In the proposed system, the bilateral grip control task is used to assess the upper limb grip strength and bilateral coordination and control performance of stroke victims to understand the correlation between bilateral grip coordination control and the evaluation scale. Tasks are designed to simultaneously assess continuously alternating bilateral grip force formation, sustained grip and grip force release (one hand contracts while the other releases, but the total grip force of both hands is continuously maintained within a range of ±10%). Grip force formation is defined as the force generated when the fingers begin gripping the metal handle (where the grip strength is twice or greater than the standard deviation of resting force) until the target grip force is achieved within the required time frame (grip strength is within ±10% of the target force). Sustained grip is defined as grip strength maintained for 3 to 5 seconds once the target force is attained (where the grip output is within ±10% of the target force and can be maintained for 3~5 seconds). Grip force release is defined as the grip strength from the time the subject begins to release the handle (where the grip strength is less than twice the standard deviation of the average sustained grip) until the hand is completely relaxed(Naik et al., 2011). The bilateral grip control task proceeds as follows: The subjects first randomly grip both handles and increase their grip with one hand (selected at random) to within the target force range and maintain this force for 3~5 seconds. The subjects then are asked to slowly release their grip while repeating the exercise with their other hand. The task is repeated three times with each hand to create an aggregate curve for each hand. This curve provides real-time visual feedback to enhance the subject’s task performance. Data analysis is then used to identify the best performance of the three tests. In addition, the task includes three different target force levels (10%, 20% and 40% of the MVC). This provides a better understanding of whether increased force can be used to significantly distinguish between younger and older subjects in terms of coordinated bilateral force control. Therefore, each subject will complete the bilateral grip control task three times for each three target force level, for a total of 9 test rounds.

1. **Clinical assessments**

In clinical or academic settings, the Fugl-Meyer Assessment is commonly-used to assess the functional recovery of stroke patients. It provides a reasonably high degree of reliability and validity for the assessment of five aspects including movement, balance, sensation, joint activity and pain. With a total score of 226, higher scores indicate better performance for the various aspects. A score between 96 and 99 indicates that the patient is only mildly affected, while a score between 85 and 95 indicates moderate movement disability, 50-84 indicates significant dysfunction and scores below 50 indicate severe disability (Sabari et al., 2005; Lin et al., 2009; Malouin et al., 1994; Duncan et al., 1983; Fugl-Meyer et al., 1980). The assessment items for this study are used to assess the hand function recovery results for stroke victims based on the upper limb function. The Motor Assessment Scale (MAS) provides good reliability and validity and is often used with the FMA in clinical studies. This scale is suitable for use to address the motor function performance of stroke victims. With a total score of 48 points, a higher score indicates better performance, based on assessment items including hand movement, advanced hand activities, upper arm function, walking, sitting to standing, balanced sitting, supine to side lying on to intact side, and supine to sitting over side of bed. The present study incorporates three of these eight items to assess stroke victims: hand movement, advanced hand activities and upper arm function (Sabari et al., 2005; Blennerhassett et al., 2008; Malouin et al., 1994). In addition, this study uses the Wolf Motor Function Test (WMFT) (Lin et al., 2009; Wolf et al., 2001; Morris et al., 2001) and the Barthel Index (BI) to evaluate patients in terms of their performance and independence in conducting daily activities.

1. **Experimental and survey process**

This study will be conducted at the Taipei Veterans General Hospital Medical Center. Prior to testing, the researchers will explain the entire experimental process to the subjects and their families, ensure that the subjects were aware of their rights and other relevant information, and secure signed informed consent for participation.

1. **Bilateral upper limb rehabilitation training**

A review of the literature finds that stroke victims suffer from unstable hemiplegic hand grip strength (Blennerhassett et al., 2006), abnormally excessive force (Hermsdorfer et al., 2003), delayed start and end of grip action (Seo et al., 2009), long reaction times for grip control (Anens et al., 2010), difficulty performing tasks quickly (Blennerhassett et al., 2006), and an inability to smoothly adjust force levels (Naik et al., 2011). Thus the proposed training model trains both the contralateral and hemiplegic hands to provide combined visual feedback for bilateral movement and neurophysical response to assist the recovery of hemiplegic limb and hand movement in stroke victims. In the first part of the training, the patient grips and releases with both hands simultaneously to provide the patient with visual feedback as both hands simultaneously relax and then tighten. The training program is written in Labview, training beginning with a target grip force of 10% MVC of the hemiplegic hand, then slowly increase and decrease the grip strength at a rate of 0.1% of MVC per second. If the subject shows significant task control improvement after training then we gradually increase the rate of grip strength change through 0.5%, 1%, 2% and 5% of MVC per second before challenging the target grip force of 20% or 40% MVC of the hemiplegic hand. This training protocol achieves bilateral training of the hemiplegic limb and helps the hemiparetic hand gradually adapt to increased grip strength (Naik et al., 2011; Renner et al., 2009).


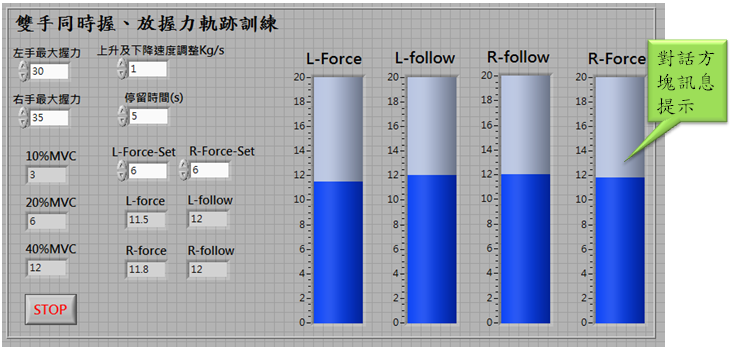


Figure: Bilateral simultaneous grip and release tracking training interface

In the second part, we track performance as the subject simultaneously grips and releases the handles with alternate hands. According to previous findings, the treatment effects of bilateral training of hands with different grip strength, inducing grip performances of the hemiplegic hand due to a grip strength ratio of less than 1:8. If the ratio is higher than 8:1 or 1:8 (i.e., 9:1 or 1:9) the bilateral exercise will result in different motion characteristics, and will not result in a bilateral coupling effect during training (Hu & Newell, 2011). Therefore, this study sets 50% MVC of the hemiplegic hand as the initial training intensity. Training begins with the hemiplegic hand generating 10% MVC and gradually increasing to 50% MVC. During this time , the contralateral hand gradually decreases its grip force from 50% MVC of the hemiplegic hand to 10%.


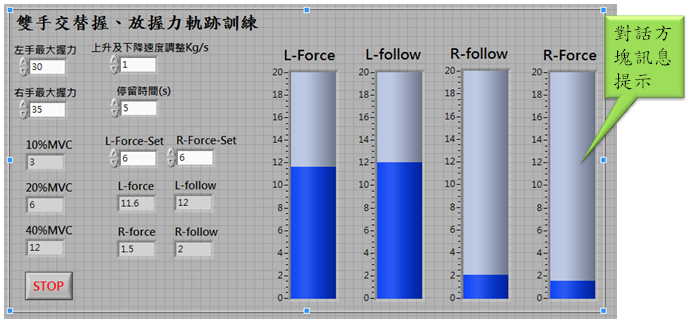


圖、雙手同時握、放之追尋握力軌跡訓練介面。

Figure: Bilateral simultaneous grip and release tracking

In addition, a meta-analysis of previous studies reporting effective functional recovery shows reported bilateral training conducted in sessions lasting 15 minutes to 2 hours, 3 to 5 times per week over a course of 2 to 8 weeks (Stewart et al., 2006). Therefore, the training sessions conducted for the present study lasted 30 minutes each time, 3 times per week over 4 weeks. Results of the proposed computer-assisted bilateral upper limb rehabilitation training will be used to determine whether the training significantly contributes to hand and limb function recovery, and the impact on the ability of stroke victims to engage in daily life activities.

1. **Statistical analysis**

SPSS version 16.0 will be used to integrate all data for parameter analysis. Patient biographical data will be described statistically. Continuous variation in the data will be addressed through a comparison in two groups using the Mann-Whitney U test, while categorical variation will be addressed through comparison in two groups using the Chi-square test. Differences in pre- and post-training performance for the groups will be analyzed using Two-way ANOVA with repeated measure. In addition, to understand the predictive value of the assessment parameters for movement recovery, we will use Spearman’s correlation coefficient analysis to explore the relationship between each assessment scale and the corresponding measurement parameter and verify its statistically significance at p<0.05.

**References**

1. Stewart KC, Cauraugh JH, Summers JJ. Bilateral movement training and stroke rehabilitation: a systematic review and meta-analysis. *J Neurol Sci.* May 15 2006;244(1-2):89-95.
2. Sabari JS, Lim AL, Velozo CA, Lehman L, Kieran O, Lai JS. Assessing arm and hand function after stroke: a validity test of the hierarchical scoring system used in the motor assessment scale for stroke. *Arch Phys Med Rehabil.* Aug 2005;86(8):1609-1615.
3. Blennerhassett JM, Carey LM, Matyas TA. Clinical measures of handgrip limitation relate to impaired pinch grip force control after stroke. *J Hand Ther.* Jul-Sep 2008;21(3):245-252; quiz 253.
4. Lin JH, Hsu MJ, Sheu CF, et al. Psychometric comparisons of 4 measures for assessing upper-extremity function in people with stroke. *Phys Ther.* Aug 2009;89(8):840-850.
5. Wolf SL, Catlin PA, Ellis M, Archer AL, Morgan B, Piacentino A. Assessing Wolf motor function test as outcome measure for research in patients after stroke. *Str*oke. Jul 2001;32(7):1635-1639.
6. Lewis GN, Byblow WD. Neurophysiological and behavioural adaptations to a bilateral training intervention in individuals following stroke. Clin Rehabil. Feb 2004;18(1):48-59.
7. Steenbergen B, Hulstijn W, de Vries A, Berger M. Bimanual movement coordination in spastic hemiparesis. Exp Brain Res. Jun 1996;110(1):91-98.
8. Morris DM, Uswatte G, Crago JE, Cook EW, 3rd, Taub E. The reliability of the wolf motor function test for assessing upper extremity function after stroke. *Arch Phys Med Rehabil.* Jun 2001;82(6):750-755.
9. Blennerhassett JM, Carey LM, Matyas TA. Grip force regulation during pinch grip lifts under somatosensory guidance: comparison between people with stroke and healthy controls. *Arch Phys Med Rehabil.* Mar 2006;87(3):418-429.
10. Hermsdorfer J, Hagl E, Nowak DA, Marquardt C. Grip force control during object manipulation in cerebral stroke. *Clin Neurophysiol.* May 2003;114(5):915-929.
11. Seo NJ, Rymer WZ, Kamper DG. Delays in grip initiation and termination in persons with stroke: effects of arm support and active muscle stretch exercise. *J Neurophysiol.* Jun 2009;101(6):3108-3115.
12. Anens E, Kristensen B, Hager-Ross C. Reactive grip force control in persons with cerebellar stroke: effects on ipsilateral and contralateral hand. Exp Brain Res. May 2010;203(1):21-30.
13. Naik SK, Patten C, Lodha N, Coombes SA, Cauraugh JH. Force control deficits in chronic stroke: grip formation and release phases. Exp Brain Res. May 2011;211(1):1-15.
14. Folstein MF, Folstein SE, McHugh PR. "Mini-mental state". A practical method for grading the cognitive state of patients for the clinician. J Psychiatr Res. Nov 1975;12(3):189-198.
15. Bohannon RW, Smith MB. Interrater reliability of a modified Ashworth scale of muscle spasticity. *Phys Ther.* Feb 1987;67(2):206-207.
16. Coombes SA, Gamble KM, Cauraugh JH, Janelle CM. Emotional states alter force control during a feedback occluded motor task. *Emotion.* Feb 2008;8(1):104-113.
17. Vaillancourt DE, Newell KM. Aging and the time and frequency structure of force output variability. *J Appl Physiol.* Mar 2003;94(3):903-912.
18. Bigland-Ritchie B, Johansson R, Lippold OC, Smith S, Woods JJ. Changes in motoneurone firing rates during sustained maximal voluntary contractions. *J Physiol.* Jul 1983;340:335-346.
19. Kent-Braun JA, Ng AV. Specific strength and voluntary muscle activation in young and elderly women and men. *J Appl Physiol.* Jul 1999;87(1):22-29.
20. Shinohara M, Li S, Kang N, Zatsiorsky VM, Latash ML. Effects of age and gender on finger coordination in MVC and submaximal force-matching tasks. *J Appl Physiol.* Jan 2003;94(1):259-270.
21. Malouin F, Pichard L, Bonneau C, Durand A, Corriveau D. Evaluating motor recovery early after stroke: comparison of the Fugl-Meyer Assessment and the Motor Assessment Scale. *Arch Phys Med Rehabil.* Nov 1994;75(11):1206-1212.
22. Duncan PW, Propst M, Nelson SG. Reliability of the Fugl-Meyer assessment of sensorimotor recovery following cerebrovascular accident. *Phys Ther.* Oct 1983;63(10):1606-1610.
23. Fugl-Meyer AR. Post-stroke hemiplegia assessment of physical properties. *Scand J Rehabil Med Suppl.* 1980;7:85-93.
24. Renner CI, Bungert-Kahl P, Hummelsheim H. Change of strength and rate of rise of tension relate to functional arm recovery after stroke. *Arch Phys Med Rehabil.* Sep 2009;90(9):1548-1556.
25. Hu X, Newell KM. Dependence of asymmetrical interference on task demands and hand dominance in bimanual isometric force tasks. *Exp Brain Res.* Feb 2011;208(4):533-541.
