# Supplementary material for: Effects of Computer-Aided Interlimb Force Coupling Training on Paretic Hand and Arm Motor Control following Chronic Stroke: A Randomized Controlled Trial
Source: PLoS One. 2015 Jul 20;10(7):e0131048. doi: 10.1371/journal.pone.0131048 (PMC4507879; doi:10.1371/journal.pone.0131048)
Supplement: S2 Protocol — (DOCX) [file pone.0131048.s009.docx]

**探討雙手握力協調訓練對於中風患者偏癱上肢復健成效**

**研究方法**

本研究將招募中風患者進行雙側上肢手部握力評估，用以分析及評估中風患者執行雙手握力協調控制的表現，並且藉由電腦輔助訓練模式，探討能否有效恢復中風患者偏癱肢體的功能恢復，以及促進雙手之間的握力協調控制能力，確認本研究應用中風患者雙側上肢手部功能訓練及評估之成效；此外，本研究將使用臨床常用之上肢功能評估量表，用以量測中風患者在訓練前後偏癱上肢功能進步情形，並且對照分析本研究開發的評估系統之指標參數進行相關因素分析，確認本系統評估參數之鑑別度，以及作為偏癱肢體功能恢復預測的預測指標之實證依據。本研究預計採取雙盲之隨機控制試驗，評估訓練前的基準值以及訓練四周後的介入成效。

1. **受試者收案及排案條件**

本研究預計徵召中風後六個月以上之中風患者60位參與本次實驗，患者來源為醫院復健科或神經內科之門診病患。受測者的收案標準規劃如下：(1)經醫師診斷確認為腦血管栓塞或出血造成之腦血管病變者；(2)患者中風次數小於三次且無其他顯著影響上肢功能之嚴重併發症者；(3)自中風算起已病發六個月以上者且病情穩定者(Naik et al., 2011)；(4)中風恢復程度已達布朗斯壯動作分期(Brunnstrom Stage)第三期以上者；(5)受測者認知功能正常可了解實驗流程及相關注意事項等內容者(MMSE≧23) (Folstein et al., 1975)；(6)修正式阿修伍爾斯氏量表(Modified Ashworth Scale)中，肩關節水平外展、內收，肘關節彎曲、伸展，手指彎曲、伸展動作肌群張力計分＜3以下者(Bohannon et al., 1987)；(7)能主動彎曲及伸直手指(Naik et al., 2011)；(8)可坐姿平衡下進行上肢運動復健訓練達半小時以上者；(9)了解實驗相關須知且同意並簽署受測者同意書者。排案標準規劃如下：(1)上肢肌肉張力過高而完全無法進行單獨運動者(Isolative Movement)者；(2)患者在視覺上有單邊偏盲(Hemianopsia)或顯著之單邊忽略(Hemineglect)而嚴重影響雙側上肢動作執行者；(3)無法控制之高血壓(190/110 mm Hg)或不穩定之心血管問題；(4)患者有伴隨前庭、小腦等疾患嚴重影響動作執行者；(5)患者有伴隨嚴重骨科或外傷等因素致使活動過程中產生疼痛等不適症狀發生者；(6)患者因中風導致認知異常或失語(aphasia)無法進行有效溝通者；(7)患者有其他神經、精神疾病或關節孿縮等相關因素，致使在實驗過程中影響上肢活動度者。

1. **主要結果量測**

本研究除量測偏癱肢體手部握力表現、雙手協調控制對稱性以及上肢穩定性運動學參數之外亦採用相關臨床量表以及評估工具作為對照評估工具使用。雙手握力協調控制評估包括1)雙手握力協調所需時間(Bilateral Handgrip Force Coordination Timing；BHF-CT)；2)雙手握力協調施力穩定值(Bilateral Handgrip Force Stable Value, BHF-SV)；3)偏癱手部動態施力穩定值(Dynamic Force Stable Value, DFSV)，以及4)雙手動態施力穩定指標(Dynamic Force Stable Index, DFSI)。除上述經由系統所量測出的雙手握力協調控制的評估參數之外，本研究亦採用相關臨床量表作為對照評估工具使用，如動作評估量表(Motor Assessment Scale; MAS) (Sabari et al., 2005; Blennerhassett et al., 2008)、傅格-梅爾評估量表(Fugl-Meyer Assessment) (Sabari et al., 2005; Lin et al., 2009)、沃夫動作功能評量(Wolf Motor Function. Test, WMFT) (Blennerhassett et al., 2008; Wolf et al., 2001; Morris et al., 2001)等量表，用以評估中風患者在雙手功能的動作表現。最後將本研究所研發之系統所量測之參數，與各臨床量表評估結果進行交叉分析，以確認本系統量測之參數，有助於評估中風患者手部功能恢復成效。

1. **雙手握力協調控制評估**

所有測試者進行本次研究分別執行2種任務，其中包括健側及偏癱手部各別、同時最大自主用力(maximal voluntary contraction; MVC)測試，以及雙手握力控制任務(bilateral hand grip control task)；其中結合視覺回饋的雙手握力控制任務包括10%、20%及40%的偏癱手部最大自主用力之目標握力，採用偏癱手部最大自主用力之目地在於使患者雙手皆能執行的任務，避免出現負向干擾，影響評估結果及降低好手的動作表現(Lewis et al., 2001; Steenbergen et al., 1996)。

最大自主用力測試主要用於研究開始時，了解受試者雙手各別握力表現的最大自主用力程度；測試方式主要要求受試者於舒適且方便施行握力姿勢下，在研究人員以口語指令“開始”及“結束”之下，盡可能以最大握力用力抓握本系統的金屬握把並維持6秒(Coombes et al., 2008; Vaillancourt et al., 2003)；本研究最大自主握力值的來源及定義，為施予間隔60秒(避免肌肉產生疲勞現象)休息時間之3次最大自主握力測試(Bigland-Ritchie et al., 1983)， 之後選取這3次最大自主握力測試中2~6秒最大握力期間所表現的最大握力值(Kent-Braun et al., 1999; Shinohara et al., 2003). 經過本測試任務確定最大自主握力值之後，即進行雙手握力控制任務。

雙手握力控制任務主要藉由本系統所建構之雙側上肢手部握力評估系統，評估中風患者雙手握力之協調控制表現，了解雙手間握力協調控制能力與評估量表間的相關性。任務操作過程包括握力產生期(grip force formation)、持續握力期(sustained grip)以及握力放鬆期(grip force release)三部分於雙手之間同時持續交替施行(一手收縮，一手放鬆，但雙手握力總力於特定的力量範圍±10%之間)；握力產生期的操作型定義是指開始驅使手指彎曲握住金屬握把產生握力(握力力量大於休息時力量的2倍標準差值以上)，直到握力力量到達目標施力之所需時間範圍(握力力量小於目標施力的±10%之內)；持續握力期的操作型定義是握力力量到達目標施力之後，維持握力輸出維持目標施力範圍內3~5秒(握力輸出位於目標施力的±10%之內並後續能維持3~5秒以上)；握力放鬆期的操作型定義是握力力量開始放鬆(握力力量小於持續握力期之力量平均的2倍標準差以下)，直到手指完全放鬆金屬握把之時間(Naik et al., 2011)。雙手握力控制任務之施測方式與過程：首先要求受試者隨機以左手或右手開始輕握金屬握把，逐漸增加握力到目標施力範圍內，之後於目標施力範圍內穩定握力輸出維持3~5秒，之後要求受試者“用力握住握把的手慢慢打開放鬆，同時另一手慢慢用力握住握把，務必使手左、右手的握力合力輸出，位於目標施力範圍內”，一旦一手完全放鬆時，另一手即於目標施力範圍內穩定握力輸出維持3~5秒，接著持續左、右手之間的握力交替進行來回3回合，過程中皆有螢幕提供左右手的握力總合曲線，此曲線用於提供注意力增強的即時視覺回饋，有助提升任務執行的表現；資料分析是取這3回合中表現最佳的一次進行分析；此外，任務測試情境包含3種不同的目標施力(10%、20%及40%最大自主用力)，如此有助於了解施力越大是否越能突顯出健康年輕及老年受試者，雙手施力的協調控制能力；因此每位受試者進行的雙手握力控制任務有3種不同的目標施力，每一不同的目標施力執行3回合測試，合計共9次的測試。

1. **臨床量表評估**

傅格-梅爾評估量表(Fugl-Meyer Assessment)為臨床醫療人員或是學術研究論文中，最常被用於評估中風患者功能恢復量測的重要評估工具，具備相當不錯的信、效度，評估內容包括運動、平衡、感覺、關節活動度及疼痛等五面向，總分226，分數越高表示患者在上述五面向內的測試項目中，有相當佳的表現；若得分96至99分之間，表示患者屬輕微動作功能障礙，85至95分之間，表示患者屬中等動作功能障礙，50至84分之間，表示患者有顯著的動作功能障礙，至於得分小於50分者，表示患者有嚴重的動作功能障礙(Sabari et al., 2005; Lin et al., 2009; Malouin et al., 1994; Duncan et al., 1983; Fugl-Meyer et al., 1980)。本研究採用的評估項目，為量表內運動面向中的上肢功能，用以了解中風患者偏癱手部功能的恢復成效。動作評估量表(Motor Assessment Scale; MAS) 是一具備良好信、效度之評估工具，並且常與傅格-梅爾評估量表(FMA)一同應用於為臨床研究中，該量表適合用於評估中風患者動作功能的表現程度，滿分48分，且得分越高表示動作功能表現越佳，評估項目包含手部動作(hand movement)、進階手部活動(advanced hand activities)、上肢功能(upper arm function)、行走(walking)、坐到站(sitting to standing)、坐姿平衡(balanced sitting)、由仰躺側翻至健側(supine to side lying on to intact side)、由仰躺轉身坐在床緣等(supine to sitting over side of bed)等八大項目，於本研究中僅評估中風患者手部動作、進階手部活動、上肢功能三部分(Sabari et al., 2005; Blennerhassett et al., 2008; Malouin et al., 1994)。此外，本研究亦使用沃夫動作功能評量(Wolf Motor Function. Test, WMFT) (Lin et al., 2009; Wolf et al., 2001; Morris et al., 2001)以及巴氏量表(Barthel Index; BI)評估患者於日常活動中，活動參與的表現能力以及生活獨立性等功能性程度。

1. **實驗及施測流程**

本研究將於台北榮總醫學中心進行實驗，在臨床試驗施行前，研究人員將先告知受測者及其家屬整體實驗流程，並詳細闡述受測者所應有之權益，確認了解相關訊息且同意者及簽署受測者同意書以參與本次實驗。

1. **雙側上肢復健訓練**

文獻回顧發現中風患者的偏癱手會出現握力不穩定(Blennerhassett et al., 2006)、產生過度不正常的施力(Hermsdorfer et al., 2003)、握力的啟動與結束會有延遲的現象(Seo et al., 2009)、執行反應性的握力控制所需時間比較長(Anens et al., 2010)、費時較長時間執行任務(Blennerhassett et al., 2006)、無法平順調整力量(Naik et al., 2011)等，因此本訓練模式採用健側手及偏癱手共同進行雙側復健訓，結合視覺回饋及雙側運動模式及其神經生理效應，誘發中風患者偏癱肢體手部功能恢復。故每次訓練內容包括二部分，首先為進行雙手同時握、放的追尋握力軌跡訓練，其次為追尋軌跡進行雙手交替握放的訓練。第一部分雙手同時握、放的追尋握力軌跡訓練，內容主要給予患者視覺回饋，控制雙手同步進行放鬆及增加握力，訓練程式以Labview撰寫，開始訓練期間之目標握力為偏癱手握力的10%MVC，以每秒增加或減少0.1%MCV之慢速增加握力及減少握力之握、放的追尋握力軌跡訓練；一旦控制進步後而逐漸改變成較快的每秒增減0.5%MVC、1%MVC、2%MVC、5%MVC，之後挑戰變化成偏癱手握力的20%MVC或40%MVC目標握力，讓偏癱肢體經由雙側訓練，增加握力變化的適應能力(Naik et al., 2011; Renner et al., 2009)。


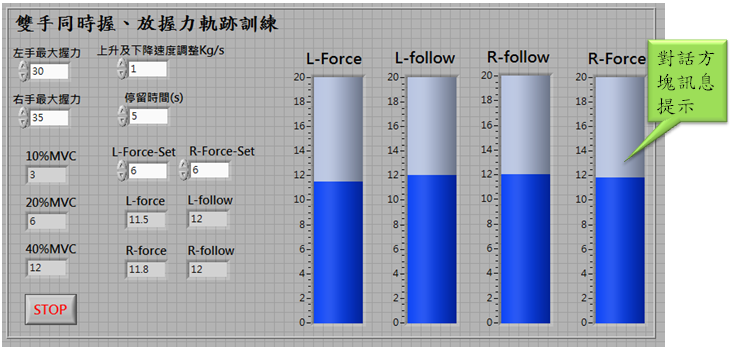


圖、雙手同時握、放之追尋握力軌跡訓練介面。

第二部分為追尋軌跡進行雙手交替握、放的訓練，根據先前研究發現，若欲藉由雙手不同握力強度的雙側訓練，誘發偏癱手部握力控制表現，則雙手間的握力差異不可達到8:1或1:8的比例，若高於8:1或1:8的比例(即9:1或1:9)，則會形成不同運動特性之雙側運動，無法達到雙手之間偶合作用(coupling effect)的誘發訓練(Hu & Newell, 2011)；因此，本研究將以偏癱手部最大握力的50%MVC作為起始訓練強度，訓練方式首先要求患者偏癱手施以10%MVC握力，健側手施以50%MVC握力，之後依訓練一的速度逐漸同時使偏癱手的10%MVC握力變成50 %MVC握力，而健側手則從50%MVC握力變成10%MVC握力。


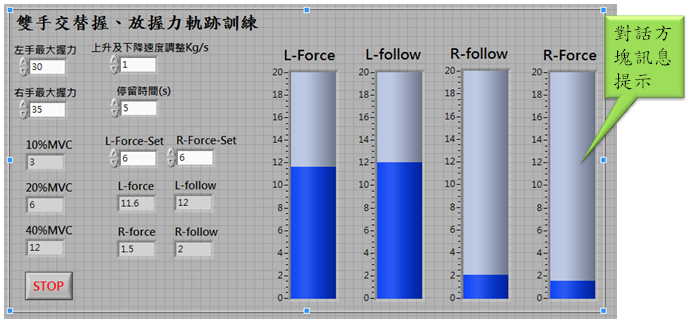


圖、雙手同時握、放之追尋握力軌跡訓練介面。

此外，統合分析性的研究發現，多數進行的雙側訓練介入為一天15分鐘到2小時之間，每周3到5天、持續進行2~8周的訓練就會有動作功能恢復的成效(Stewart et al., 2006)，因此，本次研究之訓練介入將進行為期四週，每週3次、每次30分鐘的復健訓練，了解經由本研究所規劃之電腦輔助雙側上肢復健訓練之後，是否對於中風患者偏癱肢體的手部及上肢功能恢復有顯著成效，及對於患者日常生活的功能影響。

1. **統計分析**

所有數據由研究者統整匯入電腦以Statistical Package of Social Science (SPSS)16.0版統計軟體進行參數分析。受測者之基本資料由描述型統計呈現，若資料屬性為連續型變異則採曼惠特尼U檢定(Mann-Whitney U test)進行兩組間比較；若資料屬性為類別型變異則卡方檢定(Chi-square test)進行兩組間比較。至於探討兩組於訓練介入前後及後續追蹤之表現差異，採重覆性變異數分析(Two-way ANOVA with repeated measure)進行統計分析。此外，為了瞭解各參數用於動作恢復之指標性評估參數，將使用Spearman’s進行相關係數分析，探討各評估量表相對於量測參數之相關驗證，統計上顯著差異值設p<0.05。

**參考文獻**

1. Stewart KC, Cauraugh JH, Summers JJ. Bilateral movement training and stroke rehabilitation: a systematic review and meta-analysis. *J Neurol Sci.* May 15 2006;244(1-2):89-95.
2. Sabari JS, Lim AL, Velozo CA, Lehman L, Kieran O, Lai JS. Assessing arm and hand function after stroke: a validity test of the hierarchical scoring system used in the motor assessment scale for stroke. *Arch Phys Med Rehabil.* Aug 2005;86(8):1609-1615.
3. Blennerhassett JM, Carey LM, Matyas TA. Clinical measures of handgrip limitation relate to impaired pinch grip force control after stroke. *J Hand Ther.* Jul-Sep 2008;21(3):245-252; quiz 253.
4. Lin JH, Hsu MJ, Sheu CF, et al. Psychometric comparisons of 4 measures for assessing upper-extremity function in people with stroke. *Phys Ther.* Aug 2009;89(8):840-850.
5. Wolf SL, Catlin PA, Ellis M, Archer AL, Morgan B, Piacentino A. Assessing Wolf motor function test as outcome measure for research in patients after stroke. *Str*oke. Jul 2001;32(7):1635-1639.
6. Lewis GN, Byblow WD. Neurophysiological and behavioural adaptations to a bilateral training intervention in individuals following stroke. Clin Rehabil. Feb 2004;18(1):48-59.
7. Steenbergen B, Hulstijn W, de Vries A, Berger M. Bimanual movement coordination in spastic hemiparesis. Exp Brain Res. Jun 1996;110(1):91-98.
8. Morris DM, Uswatte G, Crago JE, Cook EW, 3rd, Taub E. The reliability of the wolf motor function test for assessing upper extremity function after stroke. *Arch Phys Med Rehabil.* Jun 2001;82(6):750-755.
9. Blennerhassett JM, Carey LM, Matyas TA. Grip force regulation during pinch grip lifts under somatosensory guidance: comparison between people with stroke and healthy controls. *Arch Phys Med Rehabil.* Mar 2006;87(3):418-429.
10. Hermsdorfer J, Hagl E, Nowak DA, Marquardt C. Grip force control during object manipulation in cerebral stroke. *Clin Neurophysiol.* May 2003;114(5):915-929.
11. Seo NJ, Rymer WZ, Kamper DG. Delays in grip initiation and termination in persons with stroke: effects of arm support and active muscle stretch exercise. *J Neurophysiol.* Jun 2009;101(6):3108-3115.
12. Anens E, Kristensen B, Hager-Ross C. Reactive grip force control in persons with cerebellar stroke: effects on ipsilateral and contralateral hand. Exp Brain Res. May 2010;203(1):21-30.
13. Naik SK, Patten C, Lodha N, Coombes SA, Cauraugh JH. Force control deficits in chronic stroke: grip formation and release phases. Exp Brain Res. May 2011;211(1):1-15.
14. Folstein MF, Folstein SE, McHugh PR. "Mini-mental state". A practical method for grading the cognitive state of patients for the clinician. J Psychiatr Res. Nov 1975;12(3):189-198.
15. Bohannon RW, Smith MB. Interrater reliability of a modified Ashworth scale of muscle spasticity. *Phys Ther.* Feb 1987;67(2):206-207.
16. Coombes SA, Gamble KM, Cauraugh JH, Janelle CM. Emotional states alter force control during a feedback occluded motor task. *Emotion.* Feb 2008;8(1):104-113.
17. Vaillancourt DE, Newell KM. Aging and the time and frequency structure of force output variability. *J Appl Physiol.* Mar 2003;94(3):903-912.
18. Bigland-Ritchie B, Johansson R, Lippold OC, Smith S, Woods JJ. Changes in motoneurone firing rates during sustained maximal voluntary contractions. *J Physiol.* Jul 1983;340:335-346.
19. Kent-Braun JA, Ng AV. Specific strength and voluntary muscle activation in young and elderly women and men. *J Appl Physiol.* Jul 1999;87(1):22-29.
20. Shinohara M, Li S, Kang N, Zatsiorsky VM, Latash ML. Effects of age and gender on finger coordination in MVC and submaximal force-matching tasks. *J Appl Physiol.* Jan 2003;94(1):259-270.
21. Malouin F, Pichard L, Bonneau C, Durand A, Corriveau D. Evaluating motor recovery early after stroke: comparison of the Fugl-Meyer Assessment and the Motor Assessment Scale. *Arch Phys Med Rehabil.* Nov 1994;75(11):1206-1212.
22. Duncan PW, Propst M, Nelson SG. Reliability of the Fugl-Meyer assessment of sensorimotor recovery following cerebrovascular accident. *Phys Ther.* Oct 1983;63(10):1606-1610.
23. Fugl-Meyer AR. Post-stroke hemiplegia assessment of physical properties. *Scand J Rehabil Med Suppl.* 1980;7:85-93.
24. Renner CI, Bungert-Kahl P, Hummelsheim H. Change of strength and rate of rise of tension relate to functional arm recovery after stroke. *Arch Phys Med Rehabil.* Sep 2009;90(9):1548-1556.
25. Hu X, Newell KM. Dependence of asymmetrical interference on task demands and hand dominance in bimanual isometric force tasks. *Exp Brain Res.* Feb 2011;208(4):533-541.
